# BRG1 Activates *PR65A* Transcription to Regulate NO Bioavailability in Vascular Endothelial Cells

**DOI:** 10.3389/fcell.2020.00774

**Published:** 2020-08-12

**Authors:** Baoyu Chen, Qianwen Zhao, Tongchang Xu, Liming Yu, Lili Zhuo, Yuyu Yang, Yong Xu

**Affiliations:** ^1^Jiangsu Key Laboratory for Molecular and Medical Biotechnology, College of Life Sciences, Nanjing Normal University, Nanjing, China; ^2^Department of Pathophysiology, Nanjing Medical University, Nanjing, China; ^3^Department of Geriatrics, The Second Affiliated Hospital of Nanjing Medical University, Nanjing, China; ^4^Institute of Biomedical Research, Liaocheng University, Liaocheng, China

**Keywords:** transcriptional regulation, vascular endothelial cells, nitric oxide, eNOS, phosphorylation, phosphatase, BRG1, PP2A

## Abstract

Vascular endothelial cells contribute to the pathogenesis of cardiovascular diseases by producing and disseminating angiocrine factors. Nitric oxide (NO), catalyzed by endothelial NO synthase (eNOS), is one of the prototypical angiocrine factors. eNOS activity is modulated by site-specific phosphorylation. We have previously shown that endothelial-specific knockdown of BRG1 in *Apoe*^–/–^ mice attenuates the development of atherosclerosis, in which eNOS-dependent NO catalysis plays an antagonizing role. Here we report that attenuation of atherogenesis in mice by BRG1 knockdown was accompanied by partial restoration of NO biosynthesis by 44% in the arteries and a simultaneous up-regulation of eNOS serine 1177 phosphorylation by 59%. Indeed, BRG1 depletion or inhibition ameliorated oxLDL-induced loss of NO bioavailability and eNOS phosphorylation in cultured endothelial cells. Further analysis revealed that BRG1 regulated eNOS phosphorylation and NO synthesis by activating the transcription of protein phosphatase 2A (PP2A) structural subunit a (encoded by *PR65A*). BRG1 interacted with ETS1, was recruited by ETS1 to the *PR65A* promoter, and cooperated with ETS1 to activate *PR65A* transcription. Finally, depletion of ETS1, similar to BRG1, repressed *PR65A* induction, normalized eNOS phosphorylation, and rescued NO biosynthesis in endothelial cells treated with oxLDL. In conclusion, our data characterize a novel transcriptional cascade that regulates NO bioavailability in vascular endothelial cells.

## Introduction

Vascular endothelial cells represent one of the most important cell populations in the maintenance of vascular homeostasis (Oettgen, 2001). Rather than an isolated and static entity, vascular endothelial cells form vibrant and dynamic communications with other cell types via paracrine or endocrine routes to regulate proliferation, migration, differentiation, apoptosis, and inflammation (Vita, 2011; Liu et al., 2012; Ren et al., 2013). Specifically, the various cues produced and disseminated by vascular endothelial cells to mediate cell–cell crosstalk are termed “angiocrine signals” to reflect their endothelial origin (Rafii et al., 2016). Nitric oxide (NO) is a prototypical and one of the best characterized angiocrine factors playing versatile roles in the pathogenesis of cardiovascular diseases (Dong et al., 2020). NO biosynthesis in vascular endothelial cells is catalyzed by endothelial NO synthase (eNOS, encoded by *NOS3*). Ample evidence (Wang et al., 2020) suggests that eNOS over-expression attenuates whereas eNOS deletion aggravates a host of cardiovascular diseases although there have been a few exceptions. For instance, Ozaki et al. have reported that eNOS over-expression accelerates atherosclerosis in *Apoe*^–/–^ mice owing to the dys-regulation of its enzymatic activity indicating that eNOS activity status, rather than its overall expression level, dictates the vascular homeostasis (Ozaki et al., 2002).

Several layers of mechanisms contribute to the modulation of eNOS activity and, by extension, NO bioavailability during the pathogenesis of cardiovascular diseases. For instance, transcription rate of the *NOS3* gene can be activated by the athero-protective laminar shear flow thus elevating eNOS levels and augmenting NO production (Kurinna and Barton, 2011). On the contrary, the lipid-lowering medication statins widely prescribed for the treatment of coronary heart disease (CHD) can stimulate eNOS activity and NO production by stabilizing *NOS3* mRNA without altering transcription (Forbes and Newsome, 2016). eNOS activity is also subject to post-translational modifications. Phosphorylation of eNOS serine residue 1177 (S1177, equivalent to murine S1176 and bovine S1179) by Akt strongly boosts its activity and NO production (Michalopoulos, 2017). Consistently, S1177 dephosphorylation by protein phosphatase 2A (PP2A) suppresses eNOS activity and NO production (Abu Rmilah et al., 2019). PP2A is a heterotrimer consisting of a scaffolding subunit, a catalytic subunit, and a regulatory subunit; the scaffolding subunit and the catalytic subunit, each containing two paralogs, form the holoenzyme (Mungrue et al., 2002). The two scaffolding paralogs are encoded by *PR65A* and *PR65B* whereas the two catalytic subunits are encoded by *PP2CA* and *PP2CB* (Agnetti et al., 2005). How these genes are transcriptionally regulated in endothelial cells by different pathogenic factors to influence eNOS activity is not fully understood.

Brahma related gene 1 (BRG1) is the core component of the mammalian SWI/SNF chromatin remodeling complex (Trotter and Archer, 2008). Mice with germline deletion of BRG1 die prematurely during embryogenesis and are characterized by widespread vessel deformation due to defective angiogenesis and vasculogenesis thus implicating BRG1 as an essential regulator of endothelial development (Bultman et al., 2000; Griffin et al., 2011). Mice with postnatal BRG1 deletion in vascular endothelial cells are phenotypically indistinguishable from their wild type littermates when housed under physiological conditions (Wiley et al., 2015). We have previously shown that BRG1 knockdown in endothelial cells, achieved through an endothelial-specific lentiviral shRNA delivery system, attenuates atherosclerosis in *Apoe*^–/–^mice (Fang et al., 2013). Mechanistically, endothelial depletion of BRG1 down-regulated the expression of cell adhesion molecules thereby disrupting leukocyte adhesion and reducing vascular inflammation. Because atherogenesis is associated with a reduction in eNOS-dependent NO emission (Ghizzoni et al., 2012), we asked whether BRG1 deficiency could rescue NO bioavailability in the atherosclerotic mice. Our data as presented here suggest that BRG1 interacts with the sequence-specific transcription factor ETS1 to activate *PR65A* transcription thus dampening eNOS activity and NO production.

## Materials and Methods

### Animals

All animal experiments were reviewed and approved by the Nanjing Medical University Ethics Committee on Humane Treatment of Experimental Animals. 8-week old, male *Apoe*^–/–^ mice were fed on a high-fat diet (HFD) for 8 weeks to induce atherosclerosis. shRNA targeting BRG1 (GCUGGAGAAGCAGCAGAAG) were cloned into an endothelium-specific expression vector (Tie2p/eas) and packaged using an endothelium-specific envelope (2.2) as previously described (Pariente et al., 2008). At week 1 and week 3, the *Apoe*^–/–^mice were injected with lentivirus via tail vein as previously described (Fang et al., 2013). These mice were divided into four groups: *Apoe*^–/–^ mice receiving the control lentivirus (shC) injection and fed a control diet (chow); *Apoe*^–/–^ mice receiving the control lentivirus (shC) injection and fed a HFD diet (Western); *Apoe*^–/–^ mice receiving the BRG1 knockdown lentivirus (shBRG1) injection and fed a control diet (chow); *Apoe*^–/–^ mice receiving the BRG1 knockdown lentivirus (shBRG1) injection and fed a HFD diet (Western).

### Cell Culture and Treatment

Human umbilical vein endothelial cells (HUVEC/EAhy926, ATCC) and human embryonic kidney cells (HEK293, Invitrogen) were maintained in DMEM (Invitrogen) supplemented with 10% fetal bovine serum (FBS, Hyclone). Human primary aortic endothelial cells (HAEC, Cambrex/Lonza) were maintained in EGM-2 media with supplements supplied by the vendor; experiments were performed in primary cells between third and sixth passages as previously described (Li et al., 2019a, b). Three separate batches of cells were used in this study.

### Plasmids, Transient Transfection, and Luciferase Assay

FLAG-tagged BRG1, V5-tagged ETS-1, HA-tagged PR65α, and promoter luciferase fusion constructs for PR65A have been previously described (Sanchez-Tillo et al., 2010; Lin et al., 2011; Haesen et al., 2016; Li et al., 2019d; Dong et al., 2020; Fan et al., 2020). Small interfering RNA (siRNA) sequences were as follows: BRG1#1, AACATGCACCAGATGCACAAG; BRG1#2, GCCCATGGAGTCCATGCAT; ETS1#1, GGAGATGGCTG GGAATTCAAACT; ETS1#2, ACUUGCUACCAUCCCGUAC. Transient transfections were performed with Lipofectamine 2000 (Invitrogen). Luciferase activities were assayed 24–48 h after transfection using a luciferase reporter assay system (Promega) as previously described (Li et al., 2019c; Liu et al., 2019; Mao et al., 2020). Experiments were routinely performed in triplicate wells and repeated at least three times.

### Protein Extraction, Immunoprecipitation, and Western Blot

Whole cell lysates were obtained by re-suspending cell pellets in RIPA buffer (50 mM Tris pH7.4, 150 mM NaCl, 1% Triton X-100) with freshly added protease inhibitor tablet (Roche) as previously described (Li et al., 2019c; Lu Y. Y. et al., 2019). Specific antibodies or pre-immune IgGs (P.I.I.) were added to and incubated with cell lysates overnight before being absorbed by Protein A/G-plus Agarose beads (Santa Cruz). Precipitated immune complex was released by boiling with 1X SDS electrophoresis sample buffer. Alternatively, FLAG-conjugated beads (M2, Sigma) were added to and incubated with lysates overnight. Precipitated immune complex was eluted with 3X FLAG peptide (Sigma). Western blot analyses were performed with anti-FLAG (Sigma, F1804), anti-V5 (Sigma, F3165), anti-β-actin (Sigma, A2228), anti-BRG1 (Santa Cruz, sc-17796), anti-ETS1 (Cell Signaling Tech, 14069), anti-PR65α (Proteintech, 15882-1), anti-PR65β (Proteintech, 12621-1), anti-PP2Cα (Proteintech, 13482-1), anti-PP2Cβ (Proteintech, 12554-1), anti-eNOS (Santa Cruz, sc-654), and anti-eNOS-Ser1177 (Santa Cruz, sc-12972).

### RNA Isolation and Real-Time PCR

RNA was extracted with the RNeasy RNA isolation kit (Qiagen). Reverse transcriptase reactions were performed using a SuperScript First-strand Synthesis System (Invitrogen) as previously described (Lv et al., 2020). Real-time PCR reactions were performed on an ABI Prism 7500 system with the following primers: *BRG1*, 5′-TCATGTTGGCGAGCTATTTCC-3′ and 5′-GGTTCCGAAGTCTCAACGATG-3′; *ETS1*, 5′-GGCAGTTT CTTCTGGAATTA-3′ and 5′-CACGGCTCAGTTTCTCATA-3′; human and mouse *NOS3*, 5′-GAAGGCTTTTGATCCC CGGGTCCTG-3′ and 5′-CAGTTCCTCCAGCCGTGTGTCCAC-3′; human *PP2CA*, 5′-CGAGTGGTAACCAAGCTGCAATCA-3′ and 5′-CGTCTACGAGGTGCTGGGTCAA-3′; mouse *Pp2ca*, 5′-GAGGGTACTACTCTGTGGAGAC-3′ and 5′-CCGGCTTTC GTGATTTCCT-3′; human *PP2CB*, 5′-ACAGCTTTAGTAG ATGGACAG-3′ and 5′-CATAAGAGATCACACATTGGG-3′; mouse *Pp2cb*, 5′-GAGGGTACTACTCTGTGGAGAC-3′ and 5′ -CCGGCTTTCGTGATTTCCT-3′; human *PR65A*, 5′-CGAGTT GCCAATGTCCGCTTCAA-3′ and 5′-CGTTCTAGGATGGGC TTGACTTCAC-3′; mouse *Pr65a*, 5′-AAGGCAGTGGA GTCCTTACG-3′ and 5′-AGGTTCCGGAAGTACTGTCG-3′; human *PR65B*, 5′-GTTGTTGGTGGCAGCTTCTC-3′ and 5′-CAGCTGGGTGTGGAATTCTT-3′; mouse *Pr65b*, 5′-TTGTT GGTGGCAGCTTCTC-3′ and 5′-TTGAGTATATGCCGCTGCT G-3′. Ct values of target genes were normalized to the Ct values of housekeeping control gene (18s, 5′-CGCGGT TCTATTTTGTTGGT-3′ and 5′-TCGTCTTCGAAACTCCGAC T-3′ for both human and mouse genes) using the ΔΔCt method and expressed as relative mRNA expression levels compared to the control group which is arbitrarily set as 1.

### Chromatin Immunoprecipitation (ChIP)

Chromatin Immunoprecipitation assays were performed as previously described (Kong et al., 2019; Liu et al., 2019; Shao et al., 2019; Weng et al., 2019; Yang et al., 2019a, b). Chromatin was cross-linked with 1% formaldehyde. Cells were incubated in lysis buffer (150 mM NaCl, 25 mM Tris pH 7.5, 1% Triton X-100, 0.1% SDS, 0.5% deoxycholate) supplemented with protease inhibitor tablet. DNA was fragmented into ~500 bp pieces using a Branson 250 sonicator. Aliquots of lysates containing 100 μg of protein were used for each immunoprecipitation reaction. Precipitated genomic DNA was amplified by real-time PCR with the following primers: human *PR65A* proximal (-200/-67), 5′-AGGCTCAAACTAGTCAAATC-3′ and 5′-AGCCAGTTTACAGGGCTGC-3′; human *PR65A* distal (-800/-638), 5′-ACAGTGAGACTCGGTCTCCAC-3′ and 5′-ACTGTAGTGCAGTGGCAGGATC-3′.

### NO Measurement

Prior to each assay, endothelial cells were switched to and maintained in the Kreb’s solution (118 mM NaCl, 4.6 mM KCl, 27.2 mM NaHCO_3_, 1.2 mM MgSO_4_, 2.5 mM CaCl_2_, 1.2 mM KH_2_PO_4_, and 11.1 mM glucose) for 1 h at 37°C. Afterward, 100 μl supernatant from each well was collected and the nitrate content was measured with a Griess reagent system (Promega).

### PP2A Activity Measurement

Protein phosphatase 2A activity was measured using tissue homogenates or cell lysates with a commercially available kit (DuoSet, R&D Systems) according to vendor’s recommendations.

### Statistical Analysis

Data are presented as mean ± SD. For experiments concerning multiple groups, one-way ANOVA with *post hoc* Scheffe analyses were performed to evaluate the differences. The differences between two (control and experimental) groups were determined by two-sided, unpaired Student’s *t*-test. *p-*values smaller than 0.05 are considered significant.

## Results

### BRG1 Regulates NO Bioavailability by Modulating eNOS Phosphorylation

**FIGURE 1:**
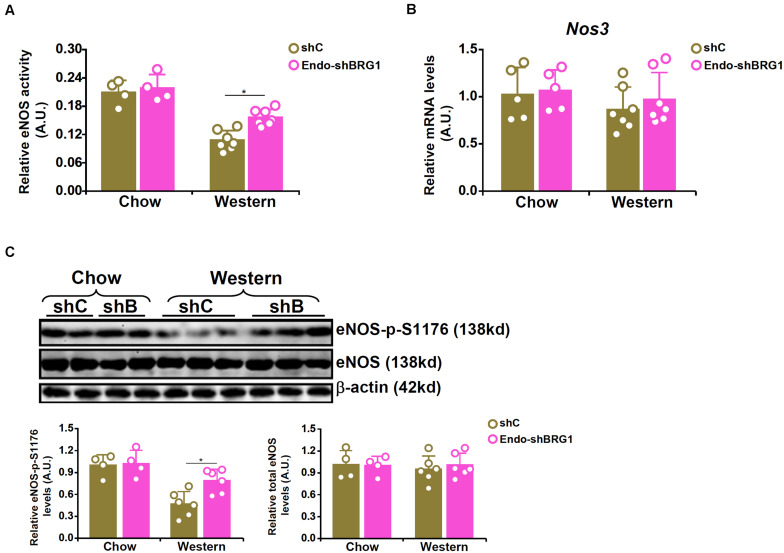
BRG1 regulates NO bioavailability by modulating eNOS phosphorylation in mice. 8-week male *Apoe*^–/–^ mice were injected with lentivirus carrying endothelial-specific BRG1 shRNA (Endo-shBRG1) or the control virus (shC) and fed a Western diet or a control diet for 8 weeks as described in “Materials and Methods.” **(A)** Aortic arteries were homogenized and NO content was examined using the Griess assay. **(B,C)** Gene expression levels in the aortic arteries were examined by qPCR and Western. *N* = 5 mice for the control diet groups and *N* = 7 mice for the Western diet groups. *p < 0.05.

We have previously reported that endothelial specific depletion of BRG1 by lentiviral mediated delivery of BRG1 shRNA (Endo-shBRG1) ameliorates the pathogenesis of atherosclerosis in *Apoe*^–/–^mice (Fang et al., 2013). Because eNOS-dependent NO production plays a key role in atherogenesis, we sought to determine whether BRG1 deficiency would influence NO bioavailability. Western blotting and immunofluorescence staining confirmed the knockdown efficiency (Supplementary Figure S1). Aortic arteries from the Endo-shBRG1/*Apoe*^–/–^ mice and the control *Apoe*^–/–^ mice were isolated and homogenized for NO quantification. As shown in Figure 1A, a considerable reduction of vascular NO content was detected in the mice fed with Western diet compared to the mice fed with the control diet. However, BRG1 deficiency significantly attenuated the decrease in NO bioavailability in the arteries (a 44% increase, shBRG1 Western diet feeding group vs. shC Western diet feeing group). Quantitative PCR (Figure 1B) and Western blotting (Figure 1C) showed that whereas overall eNOS expression in the vessels were comparable among the different groups of mice (a 12% increase was observed in the shBRG1 Western diet feeding group compared to the shC Western diet feeing group, which was not statistically significant), phosphorylation of eNOS serine 1177 (S1177) was down-regulated in the atherosclerotic mice compared to the control mice. BRG1 deficiency in endothelial cells partially restored eNOS S1177 phosphorylation (a 59% increase, shBRG1 Western diet feeding group vs. shC Western diet feeing group).

**FIGURE 2:**
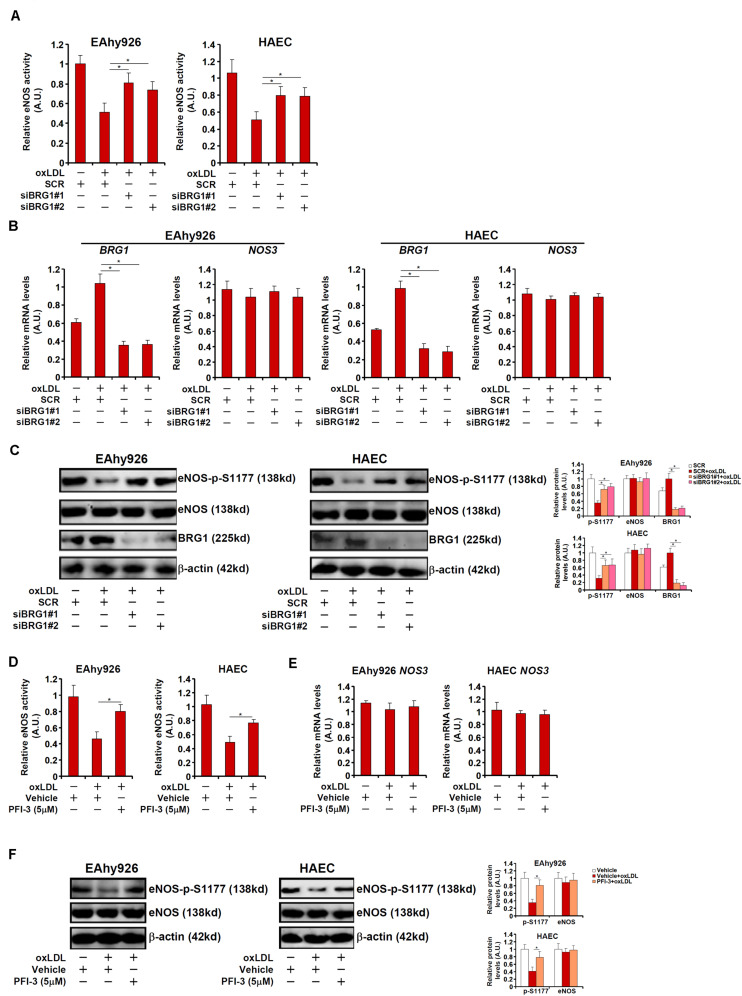
BRG1 regulates NO bioavailability by modulating eNOS phosphorylation in cultured endothelial cells. **(A–C)** EAhy926 cells and HAECs were transfected with siRNA targeting BRG1 or scrambled siRNA (SCR) followed by treatment with oxLDL (10 μg/ml) for 24 h. NO content in the culture media was examined by the Griess assay. Gene expression levels were examined by qPCR and Western. **(D–F)** EAhy926 cells and HAECs were treated with oxLDL (10 μg/ml) in the presence or absence of PFI-3 (5 μM) for 24 h. NO content in the culture media was examined by the Griess assay. Gene expression levels were examined by qPCR and Western. *p < 0.05.

We next verified the hypothesis that BRG1 may regulate NO synthesis by modulating eNOS phosphorylation in cultured endothelial cells. Stimulation with oxLDL, a known risk factor for atherosclerosis, in immortalized endothelial cells (EAhy926) and primary human aortic endothelial cells (HAECs) led to an increase in BRG1 expression as early as 12 h after the treatment and a simultaneous decrease of eNOS S1177 phosphorylation that lagged slightly behind at 24 h (Supplementary Figure S2). Exposure of EAhy926 cells and HAECs to oxLDL resulted in down-regulation of NO production whereas BRG1 knockdown by siRNAs restored NO production (Figure 2A, siBRG1#1 vs. SCR, a 57% increase in EAhy926 cells and a 56% increase in HAECs; siBRG1#2 vs. SCR, a 43% increase in EAhy926 cells and a 54% increase in HAECs). QPCR (Figure 2B) and Western blotting (Figure 2C) confirmed that oxLDL treatment suppressed eNOS S1177 phosphorylation without affecting eNOS expression; BRG1 knockdown largely normalized eNOS S1177 phosphorylation. It has been previously reported that oxLDL treatment propels eNOS to leave the caveolae compartment (Blair et al., 1999); BRG1 knockdown did not impact eNOS translocation (Supplementary Figure S3). Similarly, BRG1 inhibition by a small-molecule compound (PFI-3) attenuated the loss of NO production and eNOS S1177 phosphorylation induced by oxLDL treatment without altering eNOS expression (Figures 2D–F; PFI-3 treatment up-regulated NO content by 72% in EAhy926 cells and by 58% in HAECs). Combined, these data suggest that BRG1 may contribute to the disruption of NO production by modulating eNOS phosphorylation.

### BRG1 Mediates PP2A Transcription to Modulate NO Production

**FIGURE 3:**
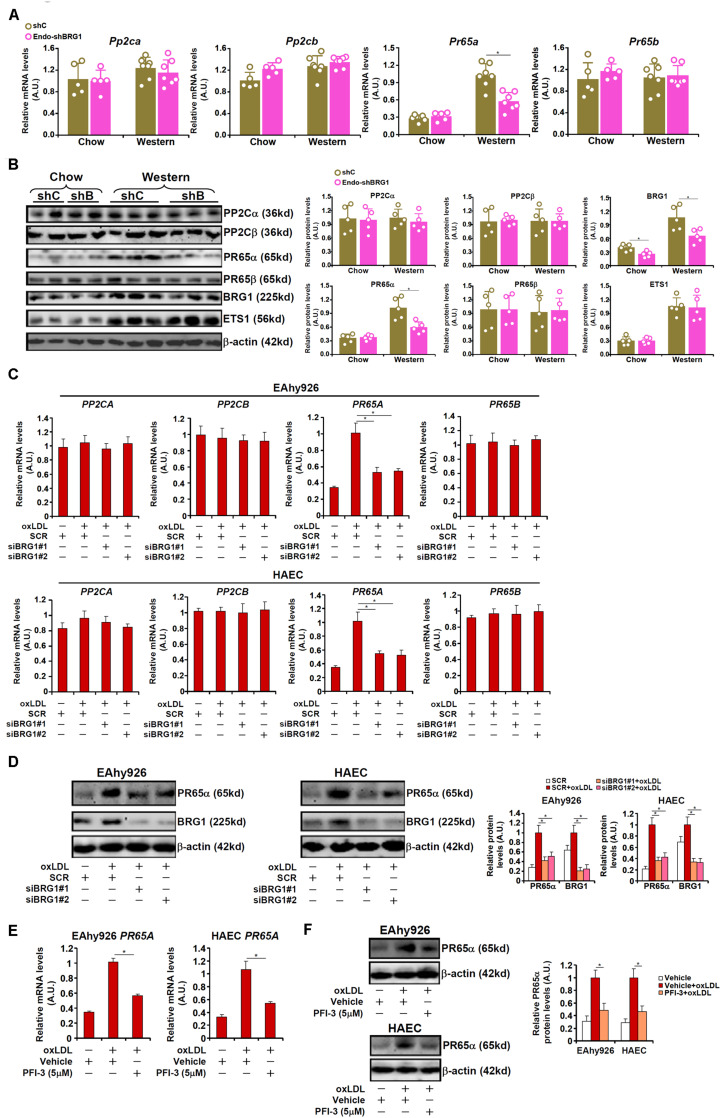
BRG1 mediates PP2A transcription. **(A,B)** 8-week male *Apoe*^–/–^ mice were injected with lentivirus carrying endothelial-specific BRG1 shRNA (Endo-shBRG1) or the control virus (shC) and fed a Western diet or a control diet for 8 weeks as described in “Materials and Methods”. Expression levels of specific genes in the aortic arteries were examined by qPCR and Western. *N* = 5 mice for the control diet groups and *N* = 7 mice for the Western diet groups. **(C,D)** EAhy926 cells and HAECs were transfected with siRNA targeting BRG1 or scrambled siRNA (SCR) followed by treatment with oxLDL (10 μg/ml) for 24 h. Gene expression levels were examined by qPCR and Western. **(E,F)** EAhy926 cells and HAECs were treated with oxLDL (10 μg/ml) in the presence or absence of PFI-3 (5 μM) for 24 h. Gene expression levels were examined by qPCR and Western. *p < 0.05.

Phosphatase 2A (PP2A) is known to remove S1177 phosphorylation and deactivate eNOS. Indeed, both PP2A and eNOS could be detected in the caveolae compartment (Supplementary Figure S4). In light of the observation that BRG1 deficiency was associated with partial restoration of S1177 phosphorylation, we hypothesized that BRG1 might regulate the expression of one or more of the PP2A subunits. Out of the four genes encoding the PP2A holoenzyme, *PR65A* expression was significantly up-regulated, at both mRNA (Figure 3A) and protein (Figure 3B) levels, in the arteries from *Apoe*^–/–^ mice fed a Western diet compared to *Apoe*^–/–^ mice fed a control diet by ∼3.6x fold; BRG1 depletion in endothelial cells attenuated *PR65A* induction (compared to the *Apoe*^–/–^ shBRG1 chow mice, a 1.8x fold increase in PR65A expression was recorded in the *Apoe*^–/–^ shBRG1 Western diet mice, equivalent to a 44% decrease compared to the *Apoe*^–/–^shC Western diet mice). Immunoprecipitation assay showed that eNOS was associated with PR65α in endothelial cells (Supplementary Figure S5). In keeping with these data, oxLDL treatment strongly enhanced the expression of *PR65A*, but not that of *PP2CA* or *PP2CB* or *PR65B*, in both EAhy926 cells and HAECs, which was antagonized by BRG1 knockdown (Figures 3C,D; siBRG1#1 vs. SCR, a 47% increase in *PR65A* expression in EAhy926 cells and a 45% increase in HAECs; siBRG1#2 vs. SCR, a 45% increase in *PR65A* expression in EAhy926 cells and a 47% increase in HAECs). BRG1 depletion also down-regulated PP2A activity as measured by phosphate release in the arteries and in cultured endothelial cells (Supplementary Figure S6). Likewise, BRG1 inhibition by PFI-3 also mitigated *PR65A* induction by oxLDL in cultured endothelial cells (Figures 3E,F; a 42% increase in *PR65A* expression in EAhy926 cells and a 49% increase in HAECs).

**FIGURE 4:**
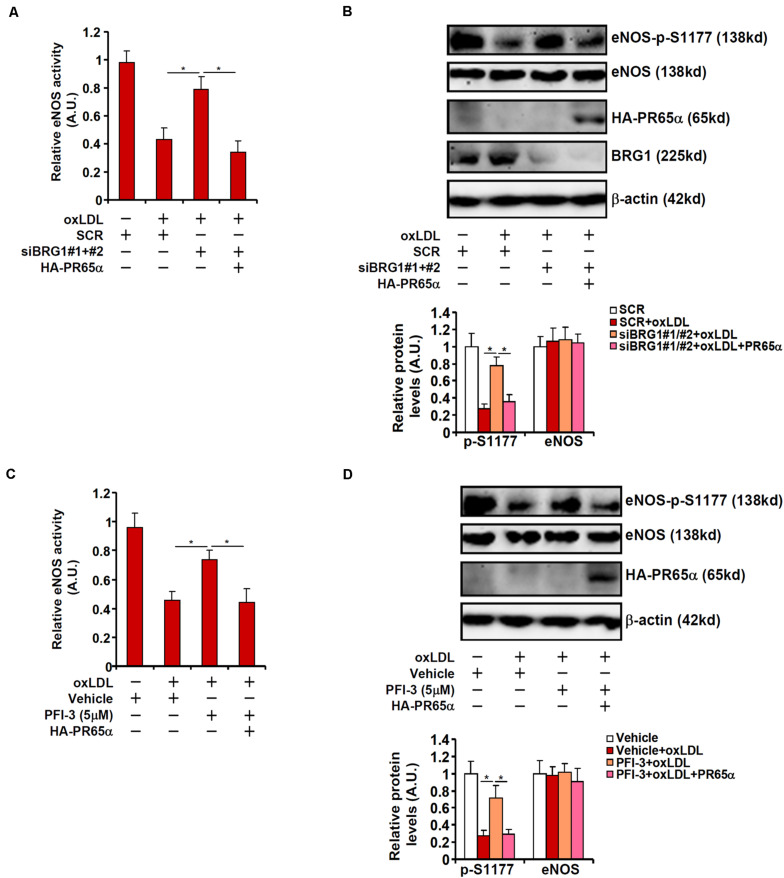
PP2A over-expression circumvents BRG1 deficiency to suppress NO production. **(A,B)** EAhy296 cells were transfected with siRNA targeting BRG1 in the presence or absence of exogenous HA-tagged PR65α followed by treatment with oxLDL (10 μg/ml) for 24 h. NO content in the culture media was examined by the Griess assay. eNOS phosphorylation was examined by Western. **(C,D)** EAhy296 cells were transfected with exogenous HA-tagged PR65α followed by treatment with oxLDL (10 μg/ml) and PFI-3 (5 μM) for 24 h. NO content in the culture media was examined by the Griess assay. eNOS phosphorylation was examined by Western. *p < 0.05.

To address the question as to whether BRG1 could regulate eNOS activity and NO bioavailability through PR65α, the following experiments were performed. As shown in Figure 4A, BRG1 knockdown attenuated suppression of eNOS activity by oxLDL treatment; forced expression of an ectopic HA-tagged PR65α enabled oxLDL to inhibit eNOS activity despite the loss of BRG1 expression. In parallel, re-introduction of PR65α into the endothelial cells circumvented BRG1 to mediate eNOS S1177 de-phosphorylation by oxLDL treatment (Figure 4B). It was also observed that PR65α over-expression was able to offset BRG1 inhibition by PFI-3 treatment and allowed oxLDL to dampen eNOS phosphorylation and NO production (Figures 4C,D). Combined, these data point to a scenario wherein BRG1 regulates PR65α expression to influence eNOS activity in endothelial cells.

## BRG1 Interacts With ETS1 to Regulate PR65A Transcription

**FIGURE 5:**
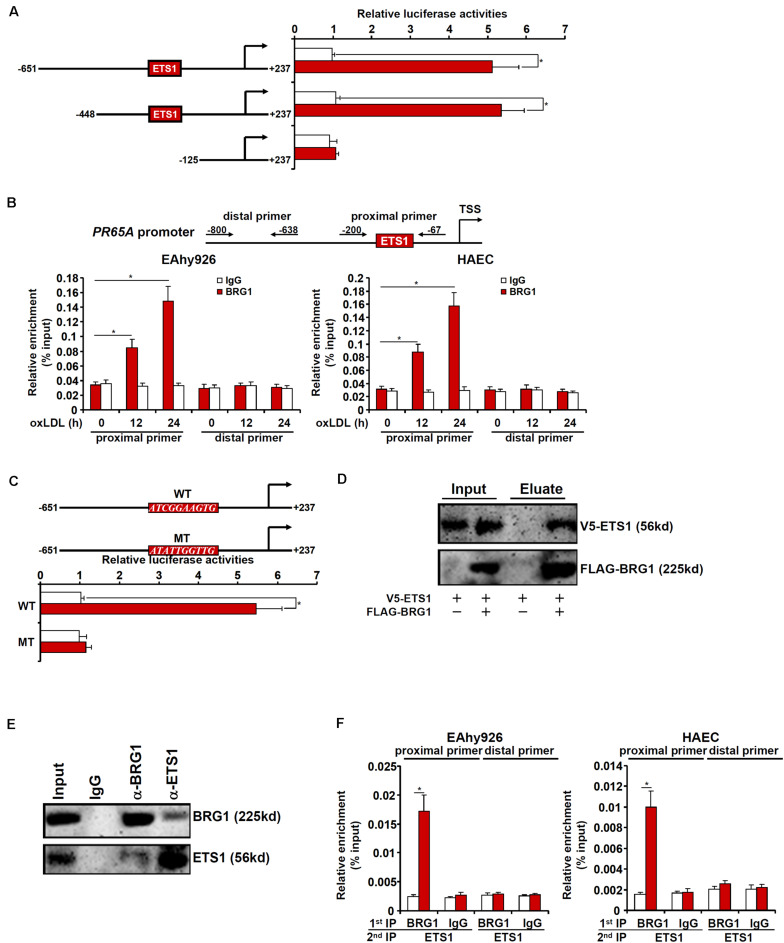
BRG1 interacts with ETS1 to regulate PR65A transcription. **(A)** *PR65A* promoter-luciferase constructs were transfected into EAhy926 cells with or without BRG1 followed by treatment with oxLDL (10 μg/ml). Luciferase activities were normalized by protein concentration and GFP fluorescence. **(B)** EAhy926 cells and HAECs were treated with oxLDL (10 μg/ml) and harvested at indicated time points. ChIP assays were performed with anti-BRG1 or IgG. **(C)** Wild type and mutant *PR65A* promoter-luciferase constructs were transfected into EAhy926 cells with or without BRG1 followed by treatment with oxLDL (10 μg/ml). Luciferase activities were normalized by protein concentration and GFP fluorescence. **(D)** FLAG-tagged BRG1 and V5-tagged EST1 were transfected into HEK293 cells. Immunoprecipitation was performed with anti-FLAG. **(E)** Nuclear lysates extracted from EAhy926 cells were immunoprecipitated with anti-BRG1. **(F)** EAhy926 cells and HAECs were with oxLDL (10 μg/ml) for 24 h. Re-ChIP assays were performed with indicated antibodies. *p < 0.05.

We next sought to determine whether and, if so, how BRG1 might directly regulate *PR65A* transcription. Luciferase reporter constructs, driven by the human *PR65A* promoter of varying lengths, were transfected into endothelial cells with or without BRG1 followed by oxLDL treatment. BRG1 over-expression plus oxLDL treatment significantly augmented the two longer *PR65A* promoter-reporter constructs (-651 and -448) but not the shortest *PR65A* promoter-reporter construct (-150), suggesting that there might be an oxLDL-responsive element between -448 and -150 of the *PR65A* promoter (Figure 5A). ChIP assay confirmed that oxLDL treatment markedly instigated the occupancies of BRG1 on the proximal *PR65A* promoter but not the distal *PR65A* promoter in a time course dependent manner (Figure 5B). A closer examination of the proximal *PR65A* promoter fragment (-448/-150) responsive to the stimulation by BRG1 over-expression plus oxLDL treatment revealed a conserved binding motif for the transcription factor ETS1; mutation of the ETS1 site completely abrogated the responsiveness of the *PR65A* promoter to BRG1 + oxLDL (Figure 5C).

We then performed a series of experiments to validate the putative ETS1-BRG1 interaction. FLAG-tagged BRG1 and V5-tagged ETS1 were transfected into HEK293 cells; immunoprecipitation with an anti-FLAG antibody simultaneously pulled down BRG1 and ETS1 (Figure 5D). More importantly, co-immunoprecipitation performed with endothelial cell lysates confirmed that endogenous BRG1 and ETS1 were in the same complex (Figure 5E). Finally, Re-ChIP assay detected a BRG1-ETS1 complex assembled on the proximal, but no the distal, *PR65A* promoter in endothelial cells exposed to oxLDL treatment (Figure 5F). Collectively, these data suggest that BRG1 might regulate *PR65A* transcription via interacting with ETS1.

### ETS1 Regulates NO Bioavailability in Endothelial Cells

**FIGURE 6:**
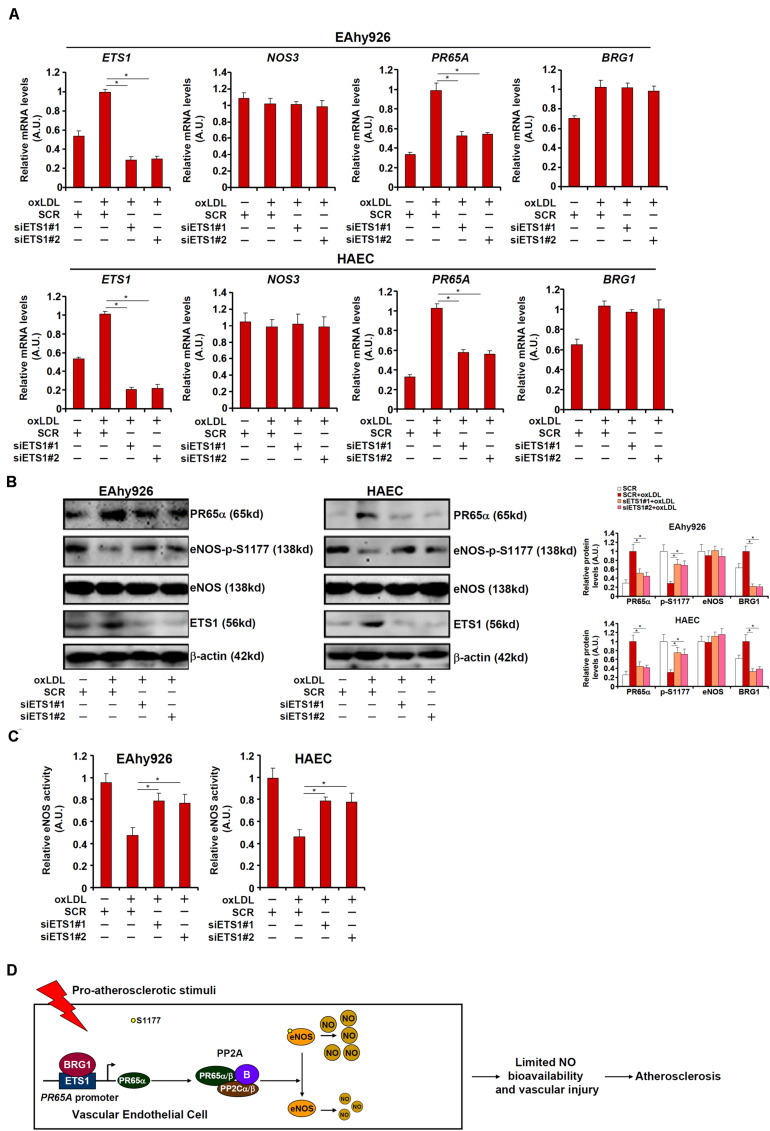
ETS1 regulates NO bioavailability in endothelial cells. **(A–C)** EAhy296 cells and HAECs were transfected with siRNA targeting ETS1 followed by treatment with oxLDL (10 μg/ml) for 24 h. NO content in the culture media was examined by the Griess assay. Gene expression levels were examined by qPCR and Western. **(D)** A schematic model. *p < 0.05.

We asked whether ETS1, like BRG1, could contribute to NO biosynthesis by modulating eNOS phosphorylation. To test this hypothesis, endogenous ETS1 was depleted by siRNAs in EAhy926 cells and HAECs. ETS1 knockdown weakened PR65α induction by oxLDL without influencing eNOS expression or BRG1 expression (Figures 6A,B; siETS1#1 vs. SCR, a 47% increase in *PR65A* expression in EAhy926 cells and a 44% increase in HAECs; siBRG1#2 vs. SCR, a 45% increase in PR65A expression in EAhy926 cells and a 46% increase in HAECs). In addition, ETS1 knockdown restored eNOS S1177 phosphorylation (Figure 6B) and alleviated the decrease in NO production by oxLDL treatment (Figure 6C). Therefore, EST1 may be a functionally relevant molecule in oxLDL-induced endothelial injury by modulating eNOS activity.

## Discussion

eNOS-mediated NO dispensing is a physiologically and pathophysiologically important process. Reduced NO bioavailability is observed in a host of cardiovascular diseases including atherosclerosis (Oemar et al., 1998), hypertension (Hermann et al., 2006), pulmonary hypertension (Zhang et al., 2016), and myocardial infarction (Jones et al., 2003). Here using both animal and cell culture models of atherosclerosis-related endothelial disorder, we provide data delineating a transcriptional cascade that regulates NO production by modulating eNOS phosphorylation. Building on a previous finding (Fang et al., 2013) that endothelial-specific knockdown of BRG1 attenuates atherosclerosis in mice by blocking leukocyte adhesion and vascular inflammation, we show here that BRG1 deficiency in endothelial cells ameliorates the loss of NO bioavailability. Mechanistically, BRG1 interacts with ETS1 to activate the transcription of *PR65A*, which encodes the scaffolding subunit of PP2A responsible for removing eNOS S1177 phosphorylation and deactivating eNOS, in endothelial cells to modulate eNOS activity. These data add to a string of recent reports by our group that implicate BRG1 as a central mediator of angiocrine signals. We have demonstrated previously that BRG1 can modulate the synthesis of a panel of secreted factors, including endothelin (ET-1) (Yang et al., 2013; Weng et al., 2015), colony stimulating factor (CSF1) (Zhang et al., 2018), interleukin 33 (IL-33) (Lu Y. et al., 2019), and tumor necrosis factor (TNF-α) (Zhang et al., 2019), in endothelial cells. A key distinction between the present finding and the previous findings, however, is that BRG1 does not seem to modulate NO synthesis by directly regulating eNOS transcription. Of note, we have previously reported that BRG1 can mediate LPS-induced suppression of eNOS activity by cooperating with the histone methyltransferase MLL1 to activate the transcription of caveolin-1 (CAV1), a well-documented inhibitor of eNOS activity (Shao et al., 2020). Because it has been documented that CAV1 expression can be activated by oxLDL treatment in endothelial cells (Sun et al., 2010; Kamada et al., 2016), a tempting possibility is that BRG1 might contribute to oxLDL-induced eNOS deactivation by simultaneously regulating multiple pathways. Another unaddressed issue is whether regulation of NO bioavailability by BRG1 observed in the pathogenesis of atherosclerosis can be extrapolated to other circumstances. PP2A-mediated dephosphorylation and inhibition of eNOS has been implicated in endothelial dysfunction associated with hypertension (Bharath et al., 2015).

Here we show that the sequence-specific transcription factor ETS1 mediates oxLDL induced reduction in NO bioavailability in endothelial cells. However, the functional relevance of ETS1 in atherogenesis is not clear at this point. EST1 clearly has a role in regulating vascular injury. Zhan et al., for instance, have reported that ETS1 contributes to Ang II induced hypertension and vascular remodeling by activating the transcription of a slew of genes involved in inflammation, apoptosis, senescence, and fibrosis (Chen et al., 2009). Further, Feng et al. have demonstrated that inhibition of ETS1 function by a dominant negative peptide, attenuates vascular remodeling in a rat model of balloon injury induced neointima formation. It should be noted that neither the Zhan study nor the Feng study examined the effect of EST1 deficiency on NO bioavailability. In fact, neither study has addressed the endothelial-specific role of EST1 in vascular injury. Of intrigue, ETS1 and BRG1 share a strikingly large number of target genes including MCP1, adhesion molecules, CTGF, and p21. Whereas ChIP-seq analysis has been performed for ETS1 in endothelial cells (Mao et al., 2020), no such dataset is yet available for BRG1. BRG1 ChIP-seq experiments performed in several other cell types such as melanoma cells (Lv et al., 2020) and epithelial cells (Haesen et al., 2016) indeed reveal a preferential enrichment of ETS1 motifs in the BRG1 peaks. This could be explained by the fact that ETS1 is a versatile transcription factor. Alternatively, it could point to a functional overlap between BRG1 and ETS1. More recently, Ye and colleagues have developed a mouse strain in which the *Ets1* allele is specifically endothelial cells driven by the *Tie2*-Cre (Oemar et al., 1998). These mice are viable, phenotypically comparable to the their wild type littermates under normal conditions but are resistant to Ang II induced cardiac fibrosis, all of which are remarkably similar to the endothelial-specific BRG1 deficient mice (Weng et al., 2015). It is yet to be determined whether the development of atherosclerosis would be affected in these mice when crossed to an *Apoe*^–/–^ background.

A few caveats need to be pointed out that may have dampened the impact of the present study. First, it is known that post-translational modifications other than phosphorylation can contribute to the modulation of eNOS activity. For instance, Erwin et al. (2005) have observed an inverse correlation between S1179 (corresponding to the human S1177) phosphorylation and S-nitrosylation of eNOS in bovine endothelial cells. Indeed, Ravi et al. (2004) have reported that eNOS S-nitrosylation, occurring primarily on cysteine 94 and cysteine 99, leads to suppression of enzymatic activity. It has been proposed by Ji and colleagues that oxLDL enhances eNOS S-nitrosylation to promote its association with the transcription factor β-catenin leading to the transcriptional activation of several genes involved in endothelial dysfunction (Wang et al., 2019). eNOS can also be reversibly acetylated and deacetylated by multiple acetyltransferases and deacetylases including SIRT1 (Mattagajasingh et al., 2007) and HDAC3 (Jung et al., 2010). Functional interactions between BRG1 and β-catenin (Li N. et al., 2019), between BRG1 and SIRT1 (Chen et al., 2019), and between BRG1 and HDAC3 (Hang et al., 2010) have been identified. It remains to be determined whether there is a crosstalk among different eNOS modifications and, if so, whether the crosstalk is mediated by BRG1. Second, we have focused on oxLDL in the present study whereas it is well documented that other pathophysiologically relevant stimuli can alter eNOS modifications. For instance, Ladurner et al. (2012) have shown that ascorbic acid, the deficiency of which is associated with increased risk of CHD (Moser and Chun, 2016), stimulates eNOS S1177 phosphorylation by suppressing PP2A activity. In addition, treatment with statin, the lipid-lowering drug widely prescribed to treat CHD, also increases eNOS S1177 phosphorylation (Rossoni et al., 2011). On the contrary, several risk factors for cardiovascular diseases, including angiotensin II (Luo et al., 2019), inflammation (Shao et al., 2020), and aging (Luo et al., 2018), all lead to down-regulation of eNOS S1177 phosphorylation and consequently its enzymatic activity. It would be of significant interest to extrapolate our findings as summarized here to different disease settings to broaden our understanding of endothelial dysfunction in the vasculature.

In summary, we describe here a novel transcriptional cascade steered by the chromatin remodeling protein BRG1 and the transcription factor ETS1 that controls eNOS activity and NO bioavailability. Future studies, exploiting more animal models preferentially with an endothelial-specific ETS1 knockout strain and genomewide profiling of shared targets for BRG1 and ETS1, would hopefully validate the proposed model (Figure 6D) and aid the development of novel therapeutic approaches in the treatment of endothelial disorders.

## Data Availability Statement

The raw data supporting the conclusions of this article will be made available by the authors, without undue reservation, to any qualified researcher.

## Ethics Statement

The animal study was reviewed and approved by the Nanjing Medical University Ethics Committee on Humane Treatment of Experimental Animals.

## Author Contributions

YX and YY conceived the project. BC, QZ, TX, LY, and YY designed and performed the experiments and collected and analyzed the data. YX wrote the manuscript. YY and LZ secured funding and provided supervision. All authors contributed to the article and approved the submitted version.

## Conflict of Interest

The authors declare that the research was conducted in the absence of any commercial or financial relationships that could be construed as a potential conflict of interest.
