# Supplementary material for: BRG1 Activates PR65A Transcription to Regulate NO Bioavailability in Vascular Endothelial Cells
Source: Front Cell Dev Biol. 2020 Aug 12;8:774. doi: 10.3389/fcell.2020.00774 (PMC7443572; doi:10.3389/fcell.2020.00774)
Supplement: Supplementary file 1 [file Image_1.pdf]

Chen BY et al: BRG1 activates PR65A transcription to regulate NO bioavailability in vascular endothelial cells
Online supplementary material

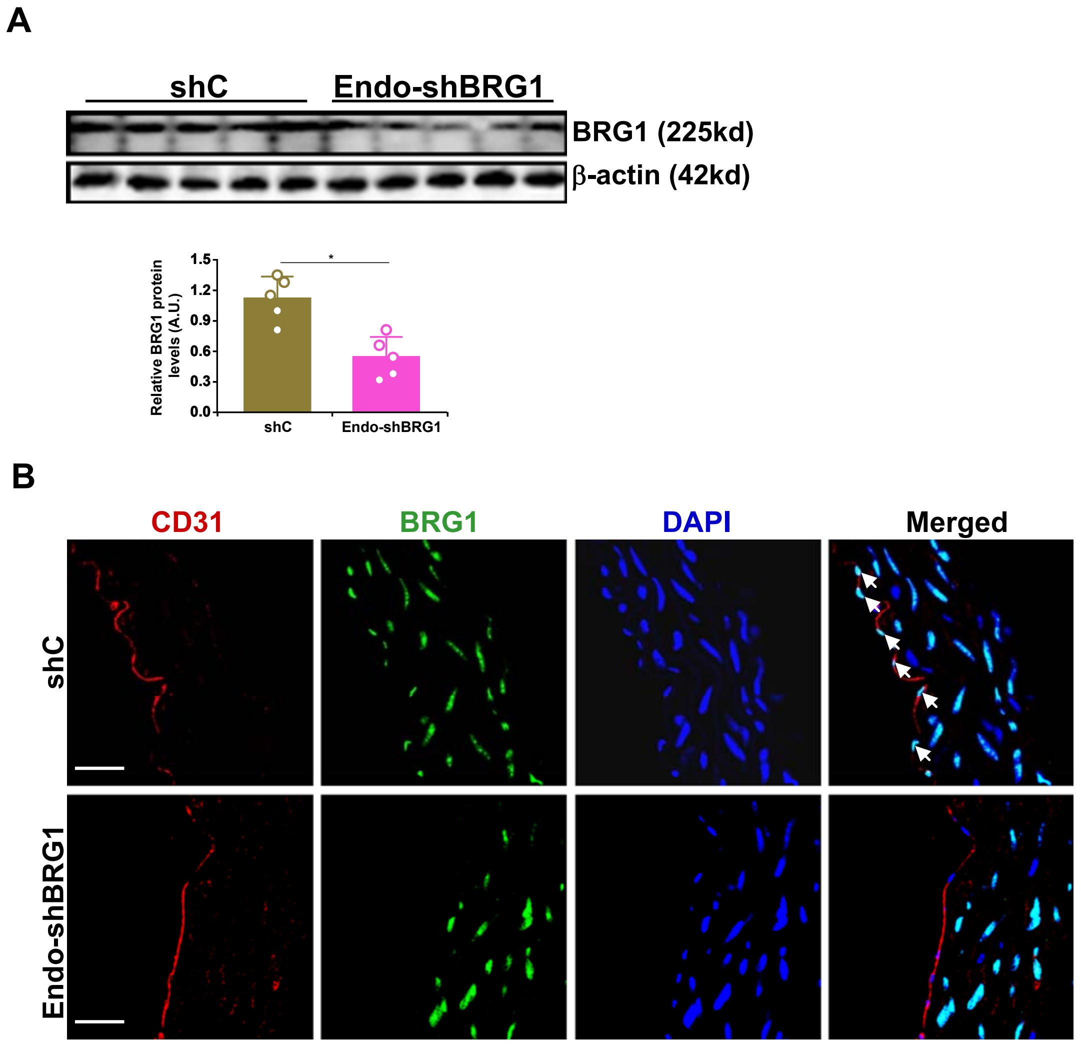

Fig.S1: 8-week male *Apoe*$^{-/-}$ mice were injected with lentivirus carrying endothelial-specific BRG1 shRNA (Endo-shBRG1) or the control virus (shC). (**A**) Whole tissue lysates were extracted from aortic arteries and BRG1 expression was examined by Western blotting. (**B**) Immunofluorescence staining was performed in paraffin sections of aortic arteries with indicated antibodies.

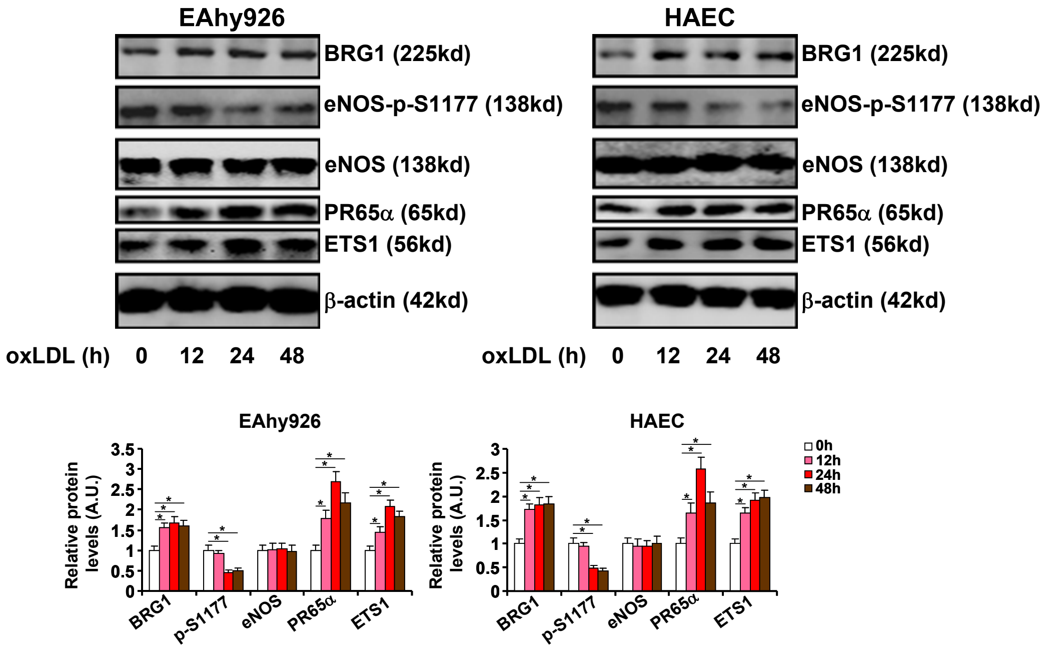

Fig.S2: EAhy296 cells and HAECs were treated with oxLDL (10μg/ml) and harvested at indicated time points. Expression levels were examined by Western blotting.

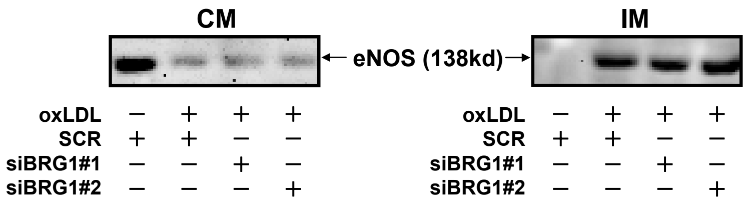

Fig.S3: EAhy926 cells were transfected with siRNA targeting BRG1 or scrambled siRNA (SCR) followed by treatment with oxLDL (10μg/ml) for 24h. Caveolae membrane (CM) and total intracellular membrane (IM) were isolated and eNOS expression was detected by Western blotting.

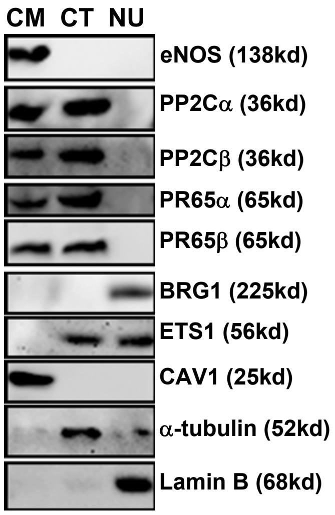

Fig.S4: EAhy926 cells were fractionated into three parts, the caveolae membrane (CM), the cytoplasm (CT), and the nucleus (NT), by standard procedure. Protein expression was detected by Western blotting.

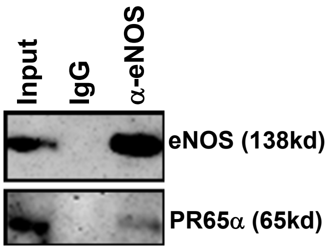

Fig.S5: Immunoprecipitation was performed with an anti-eNOS antibody using whole cell lysates extracted from EAhy926 cells.

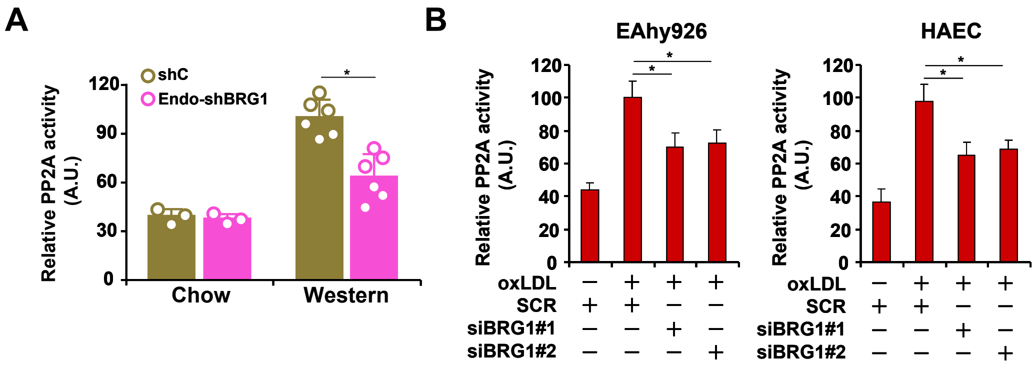

Fig.S6: (**A**) 8-week male *Apoe*$^{-/-}$ mice were injected with lentivirus carrying endothelial-specific BRG1 shRNA (Endo-shBRG1) or the control virus (shC) and fed a Western diet or a control diet for 8 weeks as described in Methods. (**B**) EAhy926 cells and HAECs were transfected with siRNA targeting BRG1 or scrambled siRNA (SCR) followed by treatment with oxLDL (10μg/ml) for 24h. PP2A activity was examined in the arteries or in endothelial cells by a commercially available kit as described in the Methods.
